# Predictive Models for Motor Outcomes From Deep Brain Stimulation in Parkinson's Disease: A Systematic Review

**DOI:** 10.1111/ejn.70644

**Published:** 2026-07-31

**Authors:** Maya Wilde, Dimitra Kiakou, Eduard Bakstein, Pavel Filip, Daniel Novak

**Affiliations:** ^1^ Department of Cybernetics, Faculty of Electrical Engineering Czech Technical University Prague Czechia; ^2^ Department of Neurology, First Faculty of Medicine Charles University Prague Czechia; ^3^ Max Planck Institute for Human Cognitive and Brain Sciences Leipzig Germany; ^4^ National Institute of Mental Health Klecany Czechia

**Keywords:** deep brain stimulation, motor outcomes, Parkinson's disease, predictive modelling

## Abstract

Deep brain stimulation (DBS) is an effective treatment for Parkinson's disease, but the extent of improvement in motor symptoms varies. A tool which accurately predicts patient outcomes based on data available pre‐surgery would be useful for clinical decision‐making and patient expectation management. Such data includes clinical and cognitive measures, neuroimaging, kinematics, functional connectivity and genetics. In this systematic review, we assess predictive models of motor outcomes from DBS. We searched the databases Web of Science, PubMed and Scopus for primary research articles that tested predictions from models based on pre‐surgical data and focused on motor outcomes from DBS for Parkinson's disease. We identified 19 studies fitting these criteria. The studies with high statistical power and generalisability use only clinical data and have limited accuracy. Studies including other types of data, such as magnetic resonance imaging, may have high accuracy but are under‐powered. Predictions are mostly limited to the first year after surgery, subthalamic nucleus‐targeted DBS and sum scores of motor performance. Following the results of the systematic search, we discuss candidate input and output variables and validation strategies to produce predictive models that are ready for translation to clinical practice. Ideally, models would predict scores for several motor domains, over a range of time after surgery, with confidence intervals. They should also be generalisable to clinics worldwide. To achieve this goal, models and datasets should be made publicly available to enable wider validation. These recommendations provide a framework to achieve predictive models for DBS outcomes that can be used clinically.

## Graphical abstract

Our systematic search uncovered 19 studies creating predictive models of motor outcomes from deep brain stimulation for Parkinson's disease. We review prediction accuracy and replicability and discuss candidate input data and the presentation of predictions.

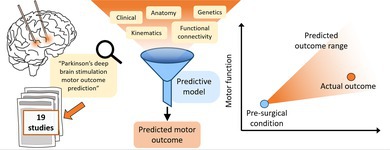

**Abbreviations**
AUC: area under the receiver operating curve
DBS: deep brain stimulation
DTI: diffusion tensor imaging
EEG: electroencephalography
fMRI: functional magnetic resonance imaging
GPi: internal globus pallidus
L‐dopa: levodopa
LASSO: least absolute shrinkage and selection operator
LGB: light gradient boosting
MDS: movement disorder society
MEG: magnetoencephalography
MRI: magnetic resonance imaging
MSE: mean squared error
NPV: negative predictive value
PKG: Parkinson's Kinetigraph
PPV: positive predictive value
SMOTE: synthetic minority oversampling technique
STN: subthalamic nucleus
SVM: support vector machine
TRIPOD: transparent reporting of a multivariable prediction model for individual prognosis or diagnosis
UPDRS: unified Parkinson's disease rating scale
VIM: ventral intermediate nucleus
VTA: volume of tissue activated

## 1 Introduction

Deep brain stimulation (DBS) has been an accepted treatment for Parkinson's disease for over 30 years (Temel et al. 2020). It typically reduces motor symptom severity and enables lower doses of pharmacological treatment. There are criteria used by clinicians to recommend against DBS treatment, either due to concerns relating to surgical outcome or low responsiveness to dopaminergic treatment (Limousin and Foltynie 2019; Defer et al. 1999; Bove, Genovese, and Moro 2022). However, amongst those who fit the criteria to receive DBS treatment, motor outcomes still vary widely. Naturally, some of this variability must arise from factors during and after the surgery, such as the surgical trajectory, the accuracy with which the target structure is reached, and postoperative programming (Wodarg et al. 2012; Wang, Younce, et al. 2025). The motivation behind predictive modelling of DBS outcomes is the idea that the remaining variability arises from factors that are present before surgery. If we can accurately estimate the degree of improvement a particular patient could expect from DBS, this would inform clinical decisions about whether to proceed with surgery or alternative treatment plans and manage patient expectations.

The ideal predictive model would use data that is both available pre‐surgery and already routinely collected. It should also be clinically interpretable, providing rationale of factors that influence its prediction. Ideally, it should have high accuracy in predicting patient outcomes: Here, we focus on motor outcomes but other measures such as overall quality of life and the reduction in medication dosage are also important to consider (Peralta et al. 2021; Mikhael et al. 2025). Further, DBS implants are intended as long‐term interventions, so the ideal model should be capable of predicting outcomes beyond the first year after surgery. The most common targets of DBS for Parkinson's are the subthalamic nucleus (STN), the internal globus pallidus (GPi) and the thalamic ventral intermediate nucleus (VIM), and considering the different connectivity of each of these structures, different factors may be more important for predicting outcomes depending on the surgical target (Lin et al. 2021; Alosaimi et al. 2022).

Useful guidelines for establishing predictive models are presented by Poldrack et al. (2020). These include using hold‐out data for testing predictions, k‐fold rather than leave‐one‐out cross‐validation, using datasets of several hundred observations and reporting multiple measures of prediction accuracy. They especially recommend reporting the area under the receiver operating curve (AUC) for classification models, and the coefficient of determination (*R* ^2^) with mean squared error for regression models. The TRIPOD statement also outlines best practice for reporting medical prediction models (Collins et al. 2015).

In this systematic review we assess the state of the field of outcome prediction for motor performance following DBS for Parkinson's disease using pre‐surgical data. Recent developments in machine learning models have led to an increase in their application in Parkinson's research (Valerio et al. 2025). Some studies have employed predictive models to optimise stimulation parameters (Mikroulis et al. 2025; Fekri Azgomi et al. 2025; Wenzel et al. 2021; Hirschmann et al. 2022), or predict outcomes using intraoperative recordings (Kostoglou et al. 2017; Park et al. 2021) or the volume of tissue activated after the electrodes are placed (Mikroulis et al. 2025; Chen, Zhu, Liu, Liu, Zhang, et al. 2022; Horn et al. 2017). However, here we specifically focus on models that are candidates for informing clinical decision‐making before surgical intervention. Following the results from the systematic search, we also provide a discussion of factors that future predictive models should consider. These include which metric of motor outcome to predict, how to present predictions to clinicians, what data types to use as inputs to predictive models, and recommendations to improve validation and reproducibility of these models. These suggestions will guide the field towards producing predictive models that can be deployed clinically.

## 2 Methods

**FIGURE 1:**
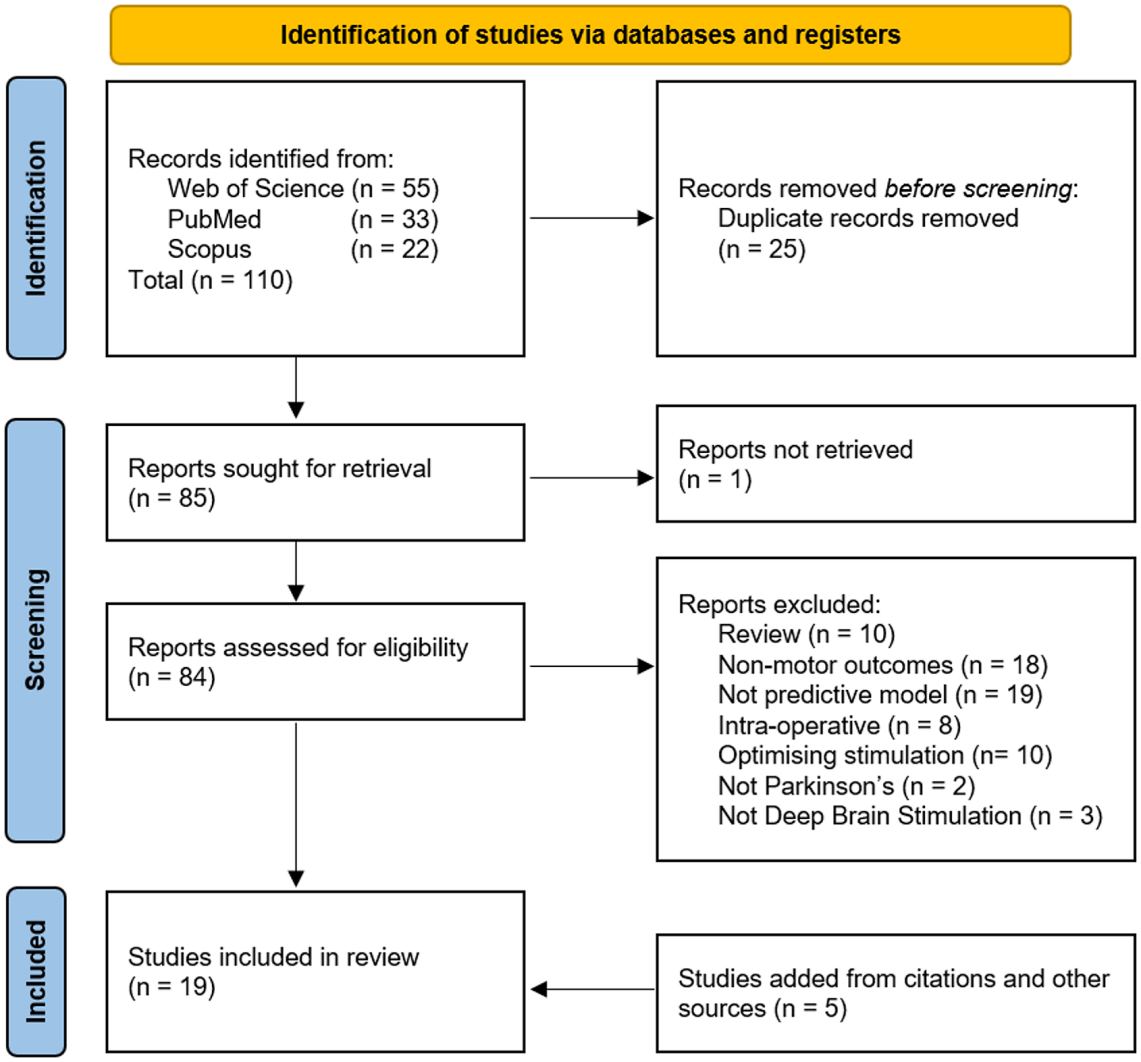
Systematic search results. 110 records were identified across three databases. After removal of duplications and exclusion based on various criteria, we identified 19 studies that create predictive models of motor outcomes from deep brain stimulation in Parkinson’s disease using pre‐operative data.

The reporting of this systematic review was guided by the standards of the 2020 PRISMA statement and checklist (Page et al. 2020). We searched the databases Web of Science (Clarivate), PubMed (NIH/NLM) and Scopus (Elsevier) on 28 November 2025. The search process is presented in Figure 1. In each database, the search terms were ‘Parkinson's’ AND ‘deep brain stimulation’ AND ‘motor outcome’ AND ‘prediction’. The search was limited to publications in English that were available for retrieval of full text. No restrictions on publication date were used.

Inclusion criteria were that studies should be primary research, focused on motor outcomes, specific to Parkinson's disease and to DBS, and presenting a predictive model based on pre‐operative information. Studies focused on metrics not available pre‐operatively such as electrode location, or using machine learning to optimise stimulation parameters were excluded. In the case of studies comparing models, some of which were solely pre‐operative and some including post‐operative information, the publication was included but only the pre‐operative models evaluated. For inclusion as predictive models, studies should report some metric of the accuracy of the predictions of their model compared with observed clinical outcomes, rather than simply reporting group level correlations (Poldrack et al. 2020; Andrews et al. 2023). For example, the coefficient of determination (*R* ^2^) between predicted and observed outcomes, or the AUC or positive and negative predictive value for binary outcomes.

## 3 Results

### 3.1 Results of the Systematic Search

After removal of duplicated and non‐available records, we screened 84 publications for inclusion eligibility (Figure 1). We excluded 10 studies which were reviews rather than primary research, and we confirmed that the scope of those reviews did not overlap with this review. We excluded 18 studies that focused on non‐motor outcomes such as quality of life, and we also excluded 19 studies that correlated pre‐operative factors with DBS outcomes without reporting accuracy of predictions from a statistical model. We excluded 8 studies that focused on intra‐operative measures and 10 which focused on optimisation of stimulation parameters following surgery. We also excluded 2 studies that were not focused on Parkinson's disease and 3 which did not concern DBS. Altogether we included 19 studies with predictive models for motor outcomes, including 5 which were harvested from citations. These are summarised in Table 1.

**TABLE 1:** Published predictive models of motor outcomes from deep brain stimulation for Parkinson's disease. Studies are grouped into those using classification or regression approaches (to indicate binary or continuous prediction types) and sorted alphabetically by first author within these categories. Studies using both types of model are in the centre. Year refers to year published, N is number of patients included.

Authors	Year	*N*	Data inputs	Model type	Prediction type	Imputation of missing data	Validation	Baseline normalisation
Classification models								
Amprimo et al. (2025 )	2025	50	Clinical data and gait parameters from motion capture	Support vector machine, AdaBoost, light gradient boosting and extreme gradient boosting	Specifically predicting gait outcomes, into two clusters based on change in gait metrics. 6 months after surgery	Not mentioned, but they did also create synthetic data for training with SMOTE	Leave‐one‐out cross validation	Outcome is not UPDRS. Change in gait metrics from pre‐ to post‐surgery are not baseline‐corrected
Habets et al. (2020 )	2020	89	Clinical only, but including cognitive measures	Logistic regression	Binary outcome based on UPDRS II, III and IV at 1 year after surgery	Yes, using random forest, only for pre‐operative values	Fivefold validation so each patient is used for testing exactly once	No, decision tree is based on absolute differences. Pre‐surgery UPDRS IV and III scores contribute the most to the model
Habets et al. (2021 )	2021	322	Clinical only, just 11 inputs	Logistic regression	Binary outcome based on UPDRS II, III and IV at 1 year after surgery	Only included patients with all data	Using previously defined model on cohorts from six centres in three continents	No, decision tree is based on absolute differences
Haliasos et al. (2024 )	2024	120	Clinical and seven radiomics features (T1 whole‐brain measures)	Random forest, logistic regression, naive Bayes, support vector machine, K‐nearest neighbours	Binary threshold is 5‐point change on UPDRS III medication‐on scores at 1 year after surgery	Excluded patients with missing MRI data	Bootstrapping: iteratively re‐dividing the dataset into training and test data	Not handled. The most strongly weighted factor is the pre‐surgery UPDRS III med‐on score
Krause et al. (2022 )	2022	105	Clinical, some MRI measures (structure volumes)	Support vector machine, logistic regression, k‐neighbours, random forest classifiers	Both binary and 3‐group, based on percentage change in UPDRS III. Time to follow‐up not clear	Impute scalar variables with mean (not for med‐on UPDRS III), label ‘missing’ for categorical	Tenfold hold‐out testing (presumably so each patient is in the test pool once)	Use percentage improvement
Liu et al. (2021 )	2021	33	10 MRI features specifically of the substantia nigra, and pre‐operative levodopa responsiveness	Logistic regression, based either on radiomics, levodopa responsiveness or both	Binary threshold of 30% improvement on MDS‐UPDRS III score 6 months after surgery, excluding tremor scores	Not mentioned	Leave‐one‐out cross‐validation	Use percentage improvement
Saudargiene et al. (2022 )	2022	34	20 features from MRI of the amygdala and hippocampus. Clinical features not included	Logistic regression, deep neural network, SVM, decision tree, linear discriminant, naive Bayes, autoencoder	Binary outcomes of Parkinson's disease composite scale scores 6 months after surgery. Very few ‘poor outcome’ patients	Not mentioned	Separated into 85% training and 15% test data. 1000 bootstrapped repetitions	Clinical features not used as inputs
Both classification and regression models								
Diao et al. (2024 )	2024	138	Clinical and MRI. Use the six top edges from structural networks and intracranial volume as inputs	Perceptron classifier, XGBoost	Predict ‘improvement percentage’ at 1 year after surgery	Not mentioned	Separated into 75% training and 25% validation set	Did not use baseline motor or L‐dopa response as an input
Geraedts et al. (2023 )	2023	75	Clinical data and Parkinson's Kinetigraph	Regression: multiple linear regression Classification: logistic regression	Compare DBS‐only and optimal therapy improvement, 1 year after surgery	Not described, but they note inherent missing data	None	Baseline correction of outcomes
Wolke et al. (2023 )	2023	429	Clinical data only, trying to minimise inputs and compare statistical methods to machine learning	Regression: linear model, Xgradient boosting tree model, support vector machine. Classification: logistic regression	Predict the absolute and relative outcome. 6–12 months after surgery	No, where data is missing for a measure, they exclude patients for that test	Data from three centres, pooled for the model. SMOTE tenfold cross‐validation used. Centre and scale data before training	Compare both absolute and relative values for improvement and L‐dopa response
Yang et al. (2023 )	2023	44	Resting fMRI low frequency fluctuations	Regression: ensemble of six models feeding into least squares regression. Classification: CatBoost	Percentage change in UPDRS III, 1 month after surgery	Not applicable (patients excluded if fMRI scans not high quality)	Tenfold cross‐validation	Did not use baseline motor or L‐dopa response as an input
Regression models								
Biesheuvel et al. (2025 )	2025	408	Clinical data	Linear regression, random forest, LASSO	Exact scores for total MDS‐UPDRS III and sub‐scores, 1 year after surgery	Imputation with Scikit‐learn ‘Iterative Imputer’	Held out a test set from the model development (~20% of data)	Predict exact outcomes to avoid measures of relative improvement
Chen, Zhu, Liu, Liu, Yuan, et al. (2022)	2022	94	Clinical data only (other models excluded as they use VTA)	Support vector machine	Predict UPDRS III percentage improvement. 1 month after surgery	Not mentioned	Held out a test set from the model development (~25% of data)	Not clear, but pre‐operative score is not used as a predictor, only medication response
Li et al. (2024)	2024	39	Resting fMRI delta controllability	Support vector regression	Change in UPDRS III score, 1 month after surgery	Excluded patients with missing data	Leave‐one‐out cross‐validation	Baseline corrected
Peralta et al. (2021 )	2021	196	Clinical data and striatal shape vectors from T1‐weighted MRI	‘PassFlow’: Artificial neural network + support vector machine, and comparison to linear regression	Predict exact values on various metrics including UPDRS III, follow‐up is up to 3 years	Previously defined artificial neural network to impute variables	50‐fold cross‐validation or leave‐one‐out cross‐validation if fewer than 50 scores available	Not clear, but predicting exact values
Shang et al. (2020 )	2020	50	Functional connectivity from fMRI (without clinical features).	Gradient boost regression tree, ordinary least squares, ridge regression, LASSO, support vector regression, extremely randomised trees.	Regression. Predict percentage change on UPDRS III. Timing of follow‐up after surgery not stated.	Not stated, presumably only patients with full fMRI data were included.	Leave‐one‐out cross‐validation.	Using percentage change, and pre‐operative score is not used as a predictor.
Wang et al. (2021 )	2021	55	Functional networks from fMRI (without clinical features)	Ridge regression, four models of different hemisphere connections	Scalar prediction of improvement in UPDRS III, 6 months after surgery	Not mentioned	Nested leave‐one‐out cross‐validation	Pre‐operative score not used as a predictor
Wang, Zhou, et al. (2025 )	2025	93	Clinical data and structural networks from diffusion tensor imaging	Multiple linear regression	Scalar prediction of change in UPDRS III, 1 month after surgery	Not mentioned	None	Baseline corrected
Younce et al. (2025 )	2025	65	Clinical, structural MRI and fMRI	Relaxed LASSO regression	Scalar prediction of change in UPDRS III, 1 year after surgery	Exclusion of patients missing data	Leave‐one‐out cross‐validation	Corrected according to Zaidel et al.

Abbreviations: CatBoost = category boosting, DBS = deep brain stimulation, fMRI = functional MRI, L‐dopa = levodopa, LASSO = least absolute shrinkage and selection operator, MDS = movement disorder society (updated UPDRS), MRI = magnetic resonance imaging, SMOTE = synthetic minority oversampling technique, SVM = support vector machine, UPDRS = unified Parkinson's disease rating scale, VTA = volume of tissue activated.

Our search is limited by publication bias, i.e., models with particularly low predictive accuracy are unlikely to be published and therefore would not be found by our search. Studies framing their research with different keywords may also have been missed by our search terms.

### 3.2 Published Predictive Models of Motor Outcomes From DBS

There are a range of studies reporting ‘predictors of motor outcome’ which simply correlate some measure with the motor outcome on a group level, rather than constructing and testing a predictive model (Poldrack et al. 2020; Andrews et al. 2023). Results from these correlative studies may inform which factors to input into a predictive model, but do not represent a testable model on their own. The studies which do create a model and report some measure of its accuracy are presented in Table 1. We have evaluated the parameters of each against the recommendations of Poldrack et al. (2020) and highlighted commendable aspects of each model.

Although we did not implement any limits on publication age, most of these studies are from the last 5 years, due to the recent increase in the use of machine learning methods (Valerio et al. 2025). Almost all studies predict short‐term outcomes, up to 12 months after surgery.

Three studies used data from more than 300 patients (Habets et al. 2021; Biesheuvel et al. 2025; Wolke et al. 2023), and six studies used data from 50 or fewer patients (Amprimo et al. 2025; Liu et al. 2021; Li et al. 2024; Saudargiene et al. 2022; Yang et al. 2023; Shang et al. 2020). Input data types used included clinical data, structural and functional MRI, and kinematics. Clinical data here refers to demographic information such as age, duration of symptoms and sex, as well as standard tests such as difference in motor scores in the presence or absence of levodopa, and ratings on Parkinson's metrics such as Hoen and Yahr scale. Almost all the studies specifically used patients with STN‐targeted DBS. Of those that did not, one study pooled patients with STN, GPi and VIM implants (Peralta et al. 2021), and another did not report the DBS target (Krause et al. 2022). Only one study created separate models for STN and GPi targeted patients (Li et al. 2024).

Across the studies presented in Table 1 there are a range of predictive models used, and several publications directly compared different model types (Wolke et al. 2023; Amprimo et al. 2025; Saudargiene et al. 2022; Krause et al. 2022; Haliasos et al. 2024). Models used for classification predictions include logistic regression, support vector machine, random forest, naïve Bayes, category boosting and k‐nearest neighbours. Models used for prediction of scalar values include support vector machine, linear regression, multiple linear regression, XGBoost and LASSO. For classification, the most common was logistic regression. For regression models, the most common were support vector machine and linear/multiple linear models.

The outcome metric predicted was most commonly the sum score of UPDRS or MDS‐UPDRS III, though some studies also looked at sub‐scores (Biesheuvel et al. 2025) or gait‐specific metrics (Amprimo et al. 2025), or incorporated MDS‐UPDRS II or IV as well (Habets et al. 2021; Habets et al. 2020). Some studies predicted the change in this score; others predicted the absolute score at follow‐up. Various approaches were taken towards missing data, including imputation or exclusion of patients with missing data (Table 1).

For prediction validation, most of the studies in Table 1 use leave‐one‐out approaches (Peralta et al. 2021; Amprimo et al. 2025; Liu et al. 2021; Shang et al. 2020; Wang et al. 2021; Younce et al. 2025; Shamir et al. 2015). Otherwise, some use bootstrapped repetitions of hold‐out data (Peralta et al. 2021; Wolke et al. 2023; Saudargiene et al. 2022; Yang et al. 2023; Krause et al. 2022; Haliasos et al. 2024), the more conservative way to do this is ensuring each sample is used in the test data exactly once (Habets et al. 2020). Two studies did not describe a validation method (Geraedts et al. 2023; Wang, Zhou, et al. 2025). Only three studies used an explicitly held‐out test set (Biesheuvel et al. 2025; Diao et al. 2024; Chen, Zhu, Liu, Liu, Yuan, et al. 2022), and we only found one publication describing validation of a previously‐defined model on entirely new data from different centres (Habets et al. 2021).

An assessment of whether studies used baseline normalisation to combat spurious associations between pre‐surgical scores or levodopa responses with outcomes is also included in Table 1 (Zaidel et al. 2010; Hope et al. 2019).

Amongst the studies found by our search, there was not consistent use of accuracy metrics. Table 2 presents the recommended metrics for classification models (area under the receiver operating characteristic curve) and regression models (coefficient of determination with root mean squared error) (Poldrack et al. 2020), as well as other commonly reported metrics (classification accuracy and positive and negative predictive value for classification models, correlation coefficient for regression models). For studies that compared many model types, we present the best‐performing ones according to the study.

**TABLE 2:** Accuracy of published predictive models. Publication order is maintained from Table 1. Where studies used a range of models for the same data, only the best performing models according to the study are reported. In two cases, RMSE is derived from reported MSE, and in another, from mean average error. The number of decimal places is maintained from the source publication, except for accuracy where it is rounded to 1 decimal place.

Classification models					Regression models				
Author	Model (if multiple)	AUC	Accuracy	PPV/NPV	Author	Model (if multiple)	*R* ^2^	*r*	RMSE
Amprimo et al. 2025	Clinical + gait		80.1%	PPV 0.64, NPV 0.91	Diao et al. 2024	XGBoost		0.67	0.235 (derived)
Habets et al. 2020		0.79	78%	PPV 0.63, NPV 0.88	Geraedts et al. 2023	PKG	0.478		
Habets et al. 2021		0.76	77%	PPV 0.62, NPV 0.79		Levodopa	0.470		
Haliasos et al. 2024	Logistic regression	0.92	88%	PPV 0.88, NPV 0.86	Wolke et al. 2023	Absolute change	0.41		
	Random forest	0.99	95%	PPV 0.96, NPV 0.94		Relative change	0.14		
Krause et al. 2022	SVM binary	0.90	81.7%		Yang et al. 2023			0.65	0.316 (derived)
	SVM multiclass		56.9%		Biesheuvel et al. 2025	Total motor score	0.3		9.1
Liu et al. 2021	Radiomics only	0.85	82%			Tremor	0.29		2.6
	Radiomics + levodopa	0.83	74%			Axial	0.31		2.5
	Levodopa alone	0.55	58%			Bradykinesia/rigidity	0.25		5.3
Saudargiene et al. 2022	Logistic regression	0.98	96.7%		Chen, Zhu, Liu, Liu, Yuan, et al. 2022	Clinical data only	0.016		
	Deep neural network	0.87	87.2%		Li et al. 2024	STN target		0.514	
Diao et al. 2024	Perceptron classifier	0.8				GPi target		0.705	
Geraedts et al. 2023	PKG	0.659			Peralta et al. 2021	3 and 6 months		~0.5	
	Levodopa	0.695				1 year		~0.4	
Wolke et al. 2023	33% change	0.72				3 years		~0.3	
	5‐point change	0.78			Shang et al. 2020	GBRT		0.65	15.52 (derived)
Yang et al. 2023		0.937	88.0%		Wang et al. 2021			0.37	
					Wang, Zhou, et al. 2025	Clinical only	0.487	0.706	0.082
						Clinical + DTI network	0.534	0.715	0.079
					Younce et al. 2025	Clinical only	0.149		14.22
						Clinical + MRI + fMRI	0.315		12.59

Abbreviations: AUC = area under the receiver operating characteristic curve, DTI = diffusion tensor imaging, fMRI = functional MRI, GBRT = gradient boost regression tree, GPi = internal globus pallidus, MRI = magnetic resonance imaging, NPV = negative predictive value, PKG = Parkinson's Kinetigraph, PPV = positive predictive value, *r* = correlation coefficient, *R* ^2^ = coefficient of determination, RMSE = root mean squared error, STN = subthalamic nucleus, SVM = support vector machine.

## 4 Discussion of Published Models

The 19 studies found by our search have each approached the task of predicting motor outcomes from DBS differently. Unfortunately, few studies have the recommended ‘several hundreds’ of patients (Poldrack et al. 2020). Indeed, in the studies presented in Tables 1 and 2, there seems to be a trend of an inverse relationship between the sample size and the model accuracy. This is difficult to quantify since the accuracy metrics are different for different approaches, but it suggests over‐fitting in studies with small sample sizes. In studies that do have larger sample sizes and even external cross‐validation, the accuracy is lower. This suggests that external validation provides more realistic performance estimates, and there is room for improvement before these tools are incorporated into clinical practice.

Very few studies predicted outcomes beyond 1 year after surgery. Naturally, data from longer‐term follow‐ups is harder to come by, but it is clinically useful to predict different time scales, and importantly, different factors are associated with motor outcomes at short and long‐term follow‐ups (Cavallieri et al. 2021; Lin et al. 2022). Notably, correlation studies have shown lack of vascular abnormalities and better scores on tests of frontal lobe performance are associated with better outcomes beyond 12 months (Limousin and Foltynie 2019; Bove, Genovese, and Moro 2022; Cavallieri et al. 2021).

Almost all of the studies exclusively used patients with STN‐targeted DBS. This is understandable given it is the most common target and therefore easier to recruit a larger cohort of patients, but a model trained to predict outcomes for one target will not necessarily translate well to another. Indeed, the one study to create a different model for each DBS target found different optimal inputs for the STN and GPi models (Li et al. 2024).

A wide range of models were used within these studies, including classification and regression approaches. Many publications compared the accuracy of different types of models, and some used both classification and regression approaches. A clear superior model does not emerge from amongst these publications, so the approach of testing several models on a dataset should continue to be employed in future studies. Beyond accuracy alone, future authors should also assess models based on their clinical interpretability (e.g., favour ‘white box’ models where factor weightings can be scrutinised) (Haliasos et al. 2024).

Missing data is a common problem in clinical datasets, and it is important that modelling studies report how they handle missing data (Collins et al. 2015), as different methods of imputation can affect conclusions in different ways (Bučková et al. 2025). Eight of the studies in Table 1 do not describe how they handle missing data. In the other studies, the authors either entirely exclude patients with missing data from analysis, or they use the recommended imputation approach of imputing input variables but not outcomes (Poldrack et al. 2020).

The recommended performance metrics for classification and regression models are AUC and *R* ^2^ with mean squared error, respectively. Several publications did not report these metrics (Table 2), which makes comparing the accuracy of models difficult. Particularly for regression models, reporting only the correlation coefficient (*r*) between predicted and observed clinical outcomes is insufficient to assess model accuracy (Poldrack et al. 2020). Studies should clearly report as many measures of prediction accuracy as possible to enable comparison between models.

Studies that have built predictive models with clinical data only have achieved some degree of prediction accuracy (Habets et al. 2021; Biesheuvel et al. 2025; Wolke et al. 2023; Habets et al. 2020). The most rigorously validated is the ‘DBS‐PREDICT’ model, which achieved 78% diagnostic accuracy between strong and weak responders in the initial patient cohort, and was then validated across 6 different centres with a diagnostic accuracy of 73%–77% (Table 1) (Habets et al. 2021; Habets et al. 2020). The other two models with large numbers of patients tested using fewer input measures or non‐machine learning statistical methods to achieve similar binary prediction accuracy (Wolke et al. 2023), or predict exact outcome scores (Biesheuvel et al. 2025). The highly weighted features in these models are consistent with those described by correlation studies, i.e., the age at surgery, duration of symptoms and levodopa‐responsiveness (Bove, Genovese, and Moro 2022). Given the large numbers of patients and strong validation, the accuracy of these studies likely represents a ceiling in the predictive ability of simple clinical data. For higher accuracy and non‐binarized outcomes it is likely necessary to add inputs from other sources. Indeed, some of the studies we identified achieved greater accuracy by incorporating neuroimaging data (Table 2) (Wang, Zhou, et al. 2025; Younce et al. 2025). However, with small sample sizes and in the absence of external validation, it is difficult to know whether this degree of accuracy would extend to other cohorts.

In summary, these 19 studies vary widely in terms of their input data, sample sizes, validation rigour and prediction accuracy. Lacking amongst these studies are predictions of outcomes beyond the first year after surgery, and cross‐validation of models on external datasets. Models for DBS targets other than the STN are also lacking, and most studies predict the sum MDS‐UPDRS III score, which does not give nuanced information about motor symptoms. Generally, adding data from other modalities to basic clinical and demographic data stands to produce models with higher accuracy, but future models need to also have higher numbers of participants to incorporate these extra factors in a statistically sound manner.

## 5 Considerations for Future Predictive Models

### 5.1 Output Variables: What to Predict?

#### 5.1.1 Measures of Motor Function

The most important decision in designing an outcome prediction model is defining what it should predict. For motor outcomes of DBS, this is most commonly the sum score on the unified Parkinson's disease rating scale (UPDRS) III (Disease MDSTF on RS for P 2003) or the movement disorder society (MDS)‐UPDRS III (Goetz et al. 2008) tests of motor function (Table 1). These tests are widely used internationally, but the sum score does not offer nuanced information about each motor symptom, which do not affect all patients equally. Tremor symptoms show the most improvement with DBS, whereas aspects like gait instability (which are mediated through non‐dopaminergic pathways; Sanjari Moghaddam et al. 2017) show less improvement (Fasano et al. 2015). This means a tremor‐dominant patient can only improve in the tremor‐related sections of the MDS‐UPDRS III, and even if their tremor symptoms are completely resolved the change in their total score may be less than that of a patient with a mixed phenotype. Breaking the questionnaire down into domains (e.g., tremor, bradykinesia, posture/gait and rigidity; Vassar et al. 2012), or using more targeted measures would enable patients and clinicians to assess which aspects of their motor activity are likely to be improved by DBS. This questionnaire also only captures a snapshot of the patient's symptoms while they are with the clinician, and does not tell us what proportion of the day the patient spends in the ‘ON’ state with good control of their movements.

**FIGURE 2:**
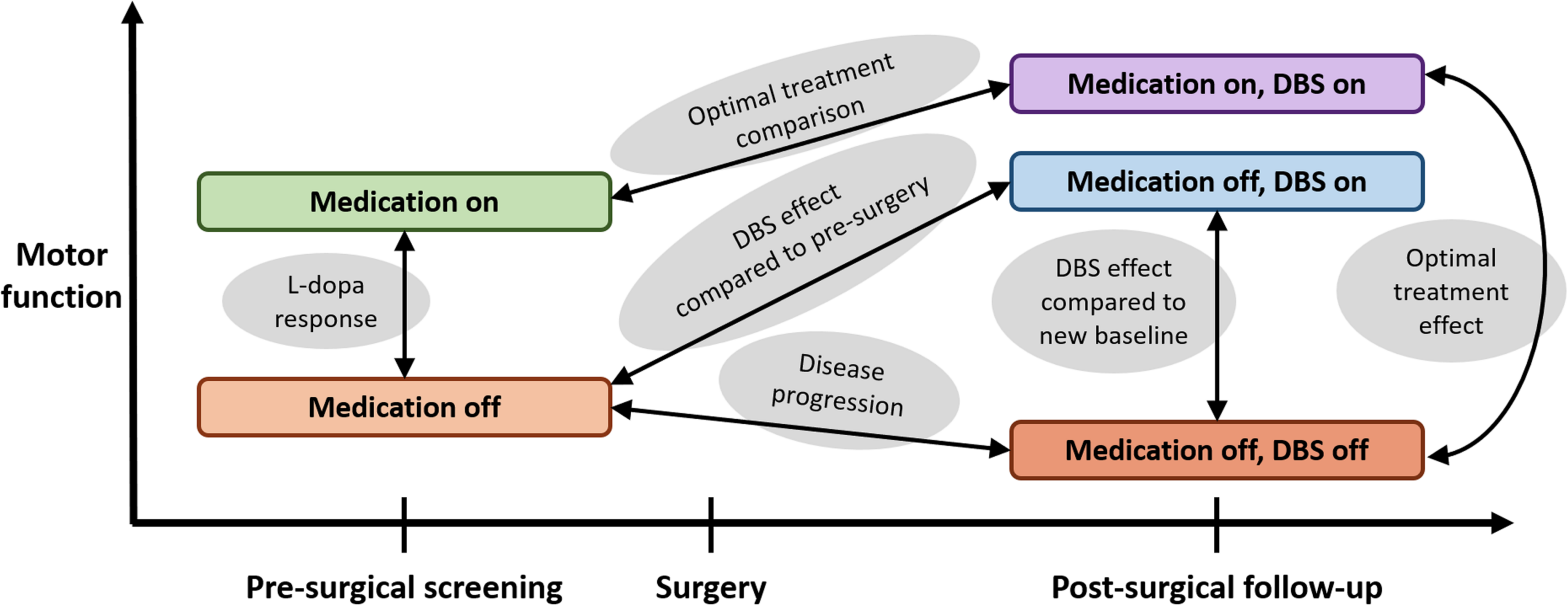
Methods to assess motor outcomes. Before surgery, the motor performance with and without levodopa treatment can be compared. After surgery, motor performance with both DBS stimulation and medication off can be compared with stimulation only or stimulation and medication combined. The improvement with DBS can therefore be described as the improvement with stimulation (and without medication) compared with either the pre‐surgery medication off condition or the post‐surgery medication and DBS off condition. Alternatively, to describe the condition the patient will live with, the optimal treatment condition before and after surgery should be compared. DBS = deep brain stimulation, L‐dopa = levodopa.

Further, the effects of DBS can be compared with pre‐surgery or stimulation off post‐surgery, and in the presence or absence of dopaminergic medication such as levodopa (Figure 2). The effect of DBS alone is measured in the absence of medication, compared with the motor performance in the absence of medication either before the surgery or after turning off the stimulation. There can be microlesion effects from surgery (Wu et al. 2025), and stimulation may produce some degree of protective effect after being switched off, so it is most typical to compare to the pre‐surgery state. This comparison gives the improvement produced by DBS compared with the patient's pre‐surgery unmedicated state. This is of interest for researchers studying the mechanism of DBS action. However, there is also an argument for comparing the medication‐on states, as these represent the actual condition the patient lives with, and are therefore an outcome of interest to the clinician and patient. Motor performance improvement with DBS stimulation combined with medication is not as dramatic compared with pre‐surgery with medication, but patients also gain benefit from subsequently reducing medication dosage and receiving consistent stimulation instead of only intermittent medication.

#### 5.1.2 Exact vs. Relative Values

A model may aim to predict the exact MDS‐UPDRS III score with DBS, but many models instead predict the difference in the motor score compared with the relevant pre‐surgery condition (Table 1). In this case, it is crucial to carefully consider how to implement the pre‐operative motor score or responsiveness to levodopa as a predictor in the model (Zaidel et al. 2010). There is a ceiling effect on outcome scores: they cannot improve beyond 0 (‘no disability’). The variance in the change in scores is therefore mainly driven by the variance in initial scores. Likewise, the variance in the absolute levodopa response is mainly driven by the severity of motor symptoms in the ‘medication off’ state. This leads to high correlation between the change in scores and either initial performance or pre‐surgery levodopa response, without them actually having good predictive value (Zaidel et al. 2010; Hope et al. 2019). A recommended method for avoiding spurious correlations is using relative rather than absolute values, e.g., dividing by the baseline medication‐off score (or using ‘percentage improvement’) (Zaidel et al. 2010). This generally retains a correlation between relative baseline and outcome scores, but not to the same extent as using the absolute value (Wolke et al. 2023; Lin et al. 2022). An assessment of whether this baseline correction is performed is included in Table 1.

#### 5.1.3 Presentation of Predictions for Clinical Use

There is also the question of how to present predicted outcomes. Some studies have used a binarized approach of good vs. poor DBS outcome, as it relates to the binary clinical decision to operate or not (Habets et al. 2021; Wolke et al. 2023; Liu et al. 2021; Saudargiene et al. 2022; Krause et al. 2022; Haliasos et al. 2024). Binarizing the outcome is suited to using a classification approach and measuring model accuracy in terms of sensitivity (identification of good responders) and specificity (identification of poor responders). However, some threshold must define what outcome is considered ‘good’, and a binary outcome does not give information on how close to this threshold a prediction is (Peralta et al. 2021; Amprimo et al. 2025). Further, these studies are generally retrospective, so patients who are not good candidates for DBS in the first place are not included. This skew makes the classification task more difficult, as the potential candidates with the worst outcomes are not present in the dataset. The division between good and poor outcomes is therefore unequal, and studies should employ methods to adjust for class imbalance (Saudargiene et al. 2022; Habets et al. 2020). Regression‐based approaches avoid this threshold difficulty and provide clinicians with an estimated outcome motor score (Biesheuvel et al. 2025) or estimated improvement with a confidence range (Chen, Zhu, Liu, Liu, Yuan, et al. 2022). This would enable them to make informed judgements, taking into account the uncertainty of the model. Future studies could also include patients who were recommended against surgery to validate that their model predicts little improvement for these patients.

As well as clinically interpretable model outputs, the factors that lead to the model producing that output should be clear to clinicians. For example, complex radiomics features from MRI data may contribute to a model with good predictive performance (Haliasos et al. 2024), but they should be described in a way that is anatomically meaningful to clinicians. Similarly, ‘white‐box’ models are preferable to ‘black‐box’ ones because the factors that influenced the decision can be examined, even if ‘black‐box’ models may achieve higher accuracy (Haliasos et al. 2024).

Further, the requirement for intensive computational power (e.g., requiring a high‐performance computing cluster) is acceptable for training the model, but should not apply to the predictive tool when in clinical use as it is unrealistic to expect clinicians to have these resources at their disposal.

**FIGURE 3:**
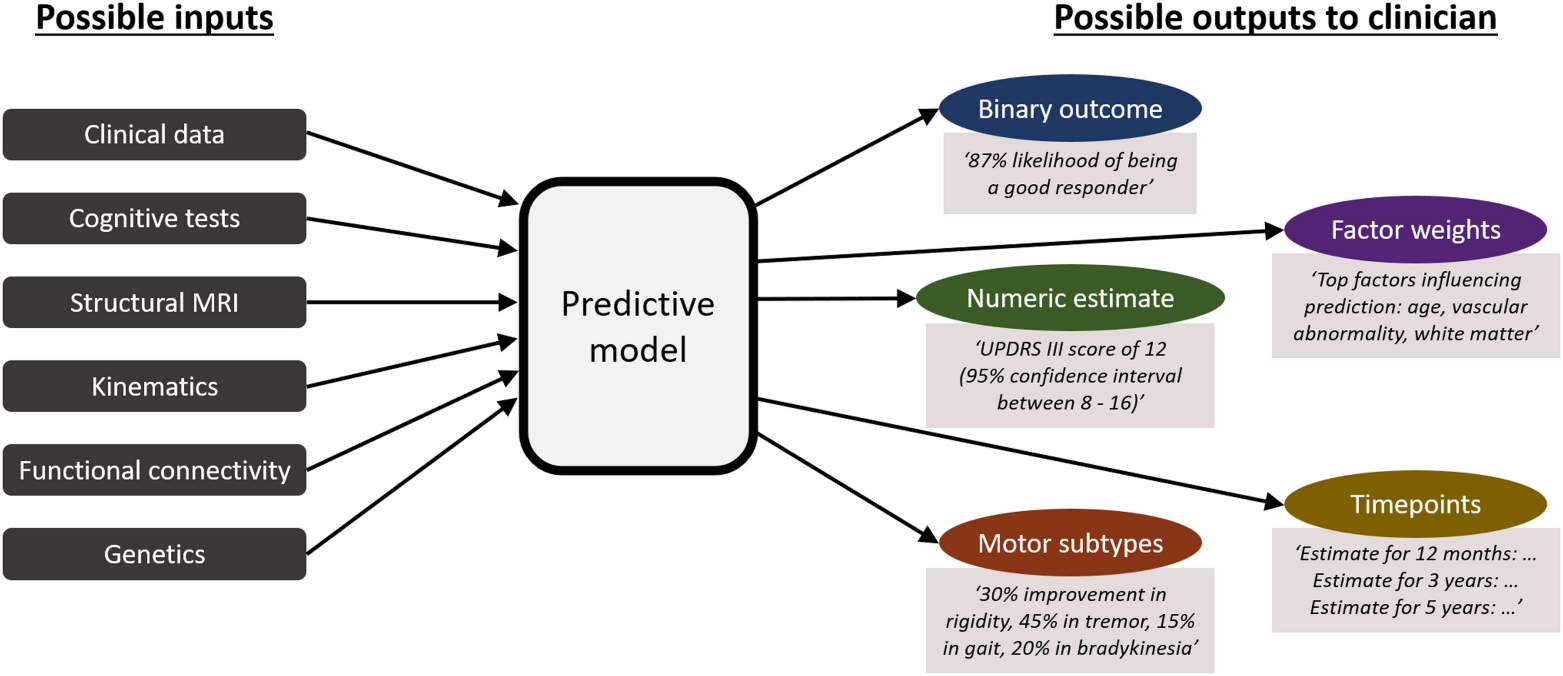
Schematic of possible predictive model. Some combination of the pre‐surgical clinical factors discussed in this review can be used as inputs, with clinical and demographic data as a starting point. Researchers should then consider how the predictions would be presented to the clinician. In the case of a binary outcome, this would be the chosen category and some measure of confidence in the classification, such as likelihood of being classified as a ‘good responder’, or the log odds between the two outcomes. For a numeric estimate, either the absolute motor questionnaire score or the relative improvement could be reported, with a range indicating the accuracy of the prediction. Output can also be broken down into subcategories of motor performance. To aid clinician understanding of the reasoning behind predictions, a summary of the most highly‐weighted factors influencing the model for that patient can be reported. A more complex model could also provide predictions for several timepoints after surgery to model the patient’s trajectory over time.

Finally, a model should be built to produce predictions for counselling individual patients, so predictions should not be restricted to group‐level estimates. An example of the type of output statements that could be useful to clinicians is presented in Figure 3.

In summary, researchers should consider how the outputs of their model can be maximally useful, accurate and interpretable to clinicians. Ideally, models should estimate outcome metrics numerically, give more nuance than the sum score of the MDS‐UPDRS III, and project beyond the first year after surgery.

### 5.2 Input Variables: Candidate Modalities of Predictive Data

#### 5.2.1 Clinical and Demographic Data

A model based solely on data collected from clinical visits during standard screening for DBS eligibility is very appealing, as the input data are readily available and clinically interpretable. As well as the clinical data models included in Table 1 (Habets et al. 2021; Biesheuvel et al. 2025; Wolke et al. 2023; Amprimo et al. 2025; Habets et al. 2020; Shamir et al. 2015), we also have information from several correlation studies (Cavallieri et al. 2021; Kim et al. 2013; Fasano et al. 2010; Bouwyn et al. 2016; Jaggi et al. 2004; Tang et al. 2022). Generally, older patients have worse outcomes, and those with longer disease duration have better outcomes (presumably due to slower disease progression) (Bove, Genovese, and Moro 2022; Lachenmayer et al. 2021). An important clinical predictor is the levodopa response before surgery (the difference between motor performance with and without medication). Patients with good responsiveness to levodopa are considered good candidates for DBS (Temel et al. 2020), but, as described in the section on exact vs. relative values, its predictive potential can be overestimated using absolute values (Zaidel et al. 2010; Hope et al. 2019). A recent meta‐analysis comparing relative improvement showed that the levodopa response before surgery correlates to the motor outcomes at 6 and 12 months after surgery, but not at 2–8 years after surgery (Lin et al. 2022). However, in one of the studies found by our search, Wolke et al. (2023) found that the absolute, but not relative, levodopa response had predictive power for the DBS outcome, suggesting that the predictive power lies in the pre‐surgical medication‐off score (Table 2).

Cardiovascular function also appears to be important to DBS outcomes: The presence of vascular abnormalities on the pre‐operative MRI scan predicts worse outcomes (Bove, Genovese, and Moro 2022; Cavallieri et al. 2021; Bove, Cavallieri, et al. 2022), and a higher cardiac washout rate is correlated with better DBS outcomes (Yamada et al. 2010).

#### 5.2.2 Cognitive Tests

Although performance on cognitive tests would typically be considered as predictors of cognitive outcomes, there is also evidence that better performance on tests of frontal lobe performance correlates with better motor outcomes from DBS (Cavallieri et al. 2021; Tang et al. 2022; Bove, Cavallieri, et al. 2022). Lower performance on cognitive tests is associated with axial symptoms, which respond less well to DBS treatment, resulting in less improvement in MDS‐UPDRS III scores (Cavallieri et al. 2021; Katz et al. 2015).

#### 5.2.3 Structural MRI

Static MRI images are already routinely collected as part of the surgical planning process, and to screen for surgical risk factors (Temel et al. 2020). Leveraging this data for DBS outcome prediction would therefore minimally impact the existing clinical workflow. A range of studies have attempted to correlate various aspects of MRI images to motor outcomes; recent reviews have shown there is little cohesion in the results (Andrews et al. 2023; Wang et al. 2022). This is partially due to inconsistencies in the outcome metric that studies attempt to predict, and also because studies do not use the same list of candidate MRI features (Wang et al. 2022).

Based on the studies found by our search, adding structural MRI data to clinical data generally improves the accuracy of predictive models (Table 2). The important features identified include the right inferior precentral area (Chen, Zhu, Liu, Liu, Yuan, et al. 2022), structural covariance between the left superior frontal gyrus and the right insular cortex, and some cerebellar regions (Diao et al. 2024), second‐order measures of whole‐brain white‐matter (Haliasos et al. 2024), left putamen volume (Krause et al. 2022) and striatal shape (Peralta et al. 2021). Wang et al. found adding DTI estimates of structural connectivity (in particular between left superior frontal gyrus and left medial frontal gyrus) to clinical data improved the performance of a predictive model for the initial outcome of DBS (Wang, Zhou, et al. 2025).

Future models should consider that the occipital cortex and cerebellum may have more predictive power than expected due to being poorly captured in previous studies (Andrews et al. 2023; Frizon et al. 2020), and that an estimate of the extent of degeneration in the substantia nigra could also be a useful predictor of DBS outcomes (Liu et al. 2021; Lönnfors‐Weitzel et al. 2016).

#### 5.2.4 Kinematics

Analysis of kinematics can produce better characterisation of motor symptoms than the standard MDS‐UPDRS III rating scale. This may involve gait assessment from video capture, or the use of wearable sensors (Youssef et al. 2026; Brognara et al. 2019). The latter enables longer‐term data collection than the brief snapshot from a clinical visit. Wearable sensors are not routinely incorporated into clinical assessment, but they are a low‐cost, easily implemented technology (Brognara et al. 2019). However, Geraedts et al. (2023) did not find that this data substantially improved predictions of MDS‐UPDRS III outcomes beyond that of standard clinical data (Table 2). It may be that kinematics are better suited to predicting motor outcomes beyond those captured by the MDS‐UPDRS III, such as daily fluctuations. Indeed, Amprimo et al. (2025) found incorporating gait data from motion capture improves prediction of change in gait parameters with DBS beyond clinical data alone (Table 2). For patients and clinicians who are specifically interested in the effects of DBS on gait, these measures give better information than the gait‐related questions on motor rating questionnaires (Shin et al. 2022).

#### 5.2.5 Functional Connectivity

Measures of functional brain activity are powerful data that represent the very network activity that DBS aims to manipulate. Collecting fMRI data is expensive and not typically involved in the standard clinical pipeline, but may be worthwhile if it proves to have strong predictive power for DBS outcomes. In our search, Younce et al. (2025) found that functional connectivity data from resting‐state fMRI improved predictive accuracy beyond clinical data alone (Table 2), and Yang et al. (2023) found predictive value in slow oscillations in resting state fMRI. Li et al. (2024) found the pre‐operative change in network controllability of the thalamus with medication was relevant to the motor outcome.

Functional connectivity could also be measured by electroencephalography (EEG) or magnetoencephalography (MEG). MEG has been used to predict motor outcomes after DBS electrode implantation (Hirschmann et al. 2022), but not pre‐operatively. EEG was not included in any of the studies we found, but it may represent a more cost‐effective measure of functional connectivity than fMRI. Measures of functional activity are therefore a fascinating avenue for outcome prediction, but there is much work to be done before we can identify factors that will generalise across the Parkinson's population.

#### 5.2.6 Genetics

There are some known Parkinson's‐related genes which may correlate to DBS outcomes. The evidence for each gene is quite scarce, which makes it difficult to assess the correlation between certain mutations and DBS outcomes (Rizzone et al. 2019). Indeed, mutations in different genes may affect the response to DBS in opposite directions: For example, GBA and PRKN tend to correlate with good motor outcomes, whereas SNCA correlates with poorer motor outcomes (Asimakidou et al. 2024). Although polygenic risk scores are an attractive candidate input to predictive models of DBS outcomes, a very large cohort would be required to find the appropriate weight to assign to genetic factors.

#### 5.2.7 Integrated Factors for Dimensionality Reduction

Adding inputs from rich data sources such as neuroimaging, kinematics and genetics to predictive models leads to a problem of having too many input factors for the number of patients (the curse of dimensionality) (Poldrack et al. 2020). In the studies we identified, statistical methods to select the most relevant features included recursive feature elimination (Liu et al. 2021), LASSO (Chen, Zhu, Liu, Liu, Yuan, et al. 2022), maximum relevancy minimum redundancy (Amprimo et al. 2025), Boruta (Amprimo et al. 2025) or random forest (Wang et al. 2021). Literature‐based feature reduction can also be performed by using information from other studies that report correlations rather than building statistical models. Another form of factor reduction could be to identify latent factors the wider data can collapse onto. Building integrative metrics such as ‘nigrostriatal integrity’ or ‘frontal connectivity’ would better capture the nature of DBS acting on interacting systems (Alosaimi et al. 2022) in a highly variable and unstable pathological environment. These metrics would ideally generalise across data from different centres and across patient‐specific pathology that leads to similar physiological outcomes.

In summary, the choice of input should be informed by how easily the data can be obtained, and how much predictive power a metric has. Clinical data should certainly continue to be used, but it should be supplemented by data from other modalities.

### 5.3 Validation and Reproducibility

A crucial aspect of developing robust models is external validation (Poldrack et al. 2020). Models built on a single cohort of data are likely to over‐fit to features that are present within that dataset but not necessarily generalisable to the wider population. This is especially true when the cohort is small. At minimum, studies should separate a training and test dataset, or use leave‐one‐out approaches for internal validation (Poldrack et al. 2020). Most of the studies found by our search use small cohorts from a single centre and do not use hold‐out testing data (Table 1). This is likely a major cause of the lack of cohesion between results from different studies. External validation such as in Habets et al. (2021) is a crucial step for confirming the performance of predictive models before they can be expected to be generalisable to clinics worldwide (Poldrack et al. 2020; Andrews et al. 2023).

External validation data can be difficult to come by, so public availability of datasets (while maintaining patient confidentiality) for cross‐validating models would greatly assist the endeavours of this field. Some projects offer to provide clinical data on request, but these do not currently include neuroimaging data (Biesheuvel et al. 2025; Hofmann et al. 2025). Factors such as MRI acquisition parameters and pharmaceutical prescription approaches can differ greatly between centres in different countries (Habets et al. 2021), so a globally useful predictive model needs to be able to generalise across these differences. Controlling for site effects will therefore also be an important step, particularly for neuroimaging data (Bayer et al. 2022). Very few of the studies we assessed made their models available for other researchers to test on their own data. As well as being important for open science, this approach would make it more feasible for cross‐validation studies to be conducted, or even better, for different published models to be compared on the same validation dataset.

## 6 Conclusions

We believe this review provides a framework for future studies to produce robustly generalisable predictive models for DBS outcomes, which are likely to be taken up for clinical use. In particular, the inputs and outputs should be clinically interpretable, and the model should be able to generalise to data from different centres. Ideally, actual motor domain‐specific scores should be predicted over a range of time‐points after DBS surgery. This will enable clinicians to make their own decision about what represents a likely ‘good’ outcome. Future studies should also follow recommendations from Poldrack et al. (2020) and the TRIPOD statement (Collins et al. 2015) to incorporate best practice and report their findings transparently. We have created a schematic in Figure 3 to illustrate hypothetical model inputs and outputs for researchers to consider when designing future predictive models. The strategies for dealing with missing data, correcting for baseline scores and validating the model should be clearly described, as many metrics of fit accuracy as possible should be reported, and models should be made available to other researchers. Data should also be made available (at least within consortia) to enable researchers to test their models on data from external centres.

## Author Contributions

**Maya Wilde:** conceptualization, writing – original draft, formal analysis, investigation, visualization. **Dimitra Kiakou:** writing – review and editing. **Eduard Bakstein:** visualization, writing – review and editing. **Pavel Filip:** conceptualization, writing – review and editing. **Daniel Novak:** funding acquisition, conceptualization, writing – review and editing.

## Funding

All the authors were supported by ERDF‐Project Brain dynamics, No. CZ.02.01.01/00/22_008/0004643. Artificial Intelligence Generated Content (AIGC) was not used in the production of this manuscript.

## Ethics Statement

The authors have nothing to report.

## Conflicts of Interest

The authors declare no conflicts of interest.

## Data Availability

Data sharing is not applicable to this article as no datasets were generated or analysed during the current study.
